# Navigating in the Gray Area of Coach-Athlete Relationships in Sports: Toward an In-depth Analysis of the Dynamics of Athlete Maltreatment Experiences

**DOI:** 10.3389/fpsyg.2022.859372

**Published:** 2022-06-02

**Authors:** Élise Marsollier, Denis Hauw

**Affiliations:** Institut des Sciences du Sport, Faculté des Sciences Sociales et Politiques, Université de Lausanne, Lausanne, Switzerland

**Keywords:** athlete lack of power, sports situation, maltreatment acceptance, maltreatment rejection, enaction

## Abstract

Several studies have revealed the abusive behaviors directed against athletes in various sports contexts, but knowledge about the processes by which the athletes realize and accept or reject maltreatment is underdeveloped. Thus, it is difficult to establish a solid scientific basis for characterizing the mechanisms of maltreatment from the athletes' perspective regarding the forms of maltreatment they endure and the impact on their performance and wellbeing. The main goals of this paper are to show how the enactive approach (including theoretical assumptions and methodological standards) can meet these challenges, as it is well-suited to (a) describe the evolving interactions between athletes and the sports situations that lead to maltreatment (i.e., navigating in the gray area of coach-athlete relationships), (b) identify those alert landmarks that help us assess the level of risk of athlete maltreatment, and (c) provide concrete guidelines to prevent and deal with sports-related maltreatment. We illustrate our approach by a case study that examines the experience of a retired high-level boxer who faced several forms of maltreatment. Our results reveal a dynamic change in the interactions between the boxer and the maltreatment situations that led her through (a) Acceptance (i.e., future-oriented positive involvement), (b) Regulation attempt (i.e., negative feelings about weight loss, exhaustion and loneliness, questioning the compromise between performance and health, acceptance and loneliness), (c) Distancing (i.e., reopening to others) and (d) Rejection (i.e., rebellion and the decision to stand up to her coach and leave). Based on our results, we present concrete guidelines to prevent and address sports-related maltreatment, with four progressive alert landmarks that help situate the athlete in the gray area of coach-athlete relationships and suggest a “timeline” of maltreatment escalation with key warnings.

## Introduction

Safeguarding refers to the measures taken to assure athletes' safety and human rights (Kerr and Stirling, 2019, p. 372), including those measures that foster the positive effects of sports participation that all athletes should benefit from. Today, maltreatment prevention appears crucial in a context in which sports participation is often accompanied by societal tensions around discrimination, cheating and other antisocial behaviors (e.g., harm, insult), as well as a disrespect for basic human rights. Indeed, recent events have demonstrated that athlete maltreatment in training and competition is not rare, but instead seems to occur more frequently than expected, with the effect of weakening the belief in the positive outcomes of sports participation (e.g., sex abuse scandal in American gymnastics, French ice skating and English soccer, Sierra Leone's women's football team; physical abuse in the Japanese Olympic women's judo team). In this article, we will use the term maltreatment as conceptualized by Fortier et al. (2020) and defined by the World Health Organization (2002, p. 59) as “All forms of physical and/or emotional abuse, sexual abuse, neglect or negligent treatment, or commercial or other exploitation, resulting in actual or potential harm to the child's health, survival, development or dignity, in the context of a relationship of responsibility, trust or power.” To counter sports-related maltreatment, safeguarding policies and procedures are generally based on prevention designs (e.g., background checks and education of coaches, implementation of responsive measures). Yet, the Safe Sport initiatives defined by the IOC are not always communicated to or applied by national sports federation leaders, who may indeed lack the financial resources and staff to deal with questions of sports-related maltreatment, despite the evidence that following these initiatives will greatly enhance their safeguarding actions (Marsollier and Verschuuren, 2022). Also, we argue that although these safeguarding procedures have been based on general human rights principles (e.g., the policies and procedures for safe and healthy sport acknowledge that all athletes have the right to be treated with respect and protected from violence), they do not sufficiently and specifically consider the situations of athletes who are faced with potential maltreatment. For example, apart from clearly unacceptable behaviors, such as striking an athlete or sexual harassment, do we truly understand the process by which some athletes accept to train with injuries or yield to other unreasonable demands, being pushed beyond their limits – and this despite the normative discourse that condemns this type of coaching behavior?

Recently, the Formula One champion Max Verstappen noted that among the reasons he is now a world champion is the harsh and disrespectful treatment he received from his father when he was a child and teen (Bielderman, 2021). The famous swimmer Fredrica Pellegrini gave a similar description of her coach Philippe Lucas's treatment (Monde, 2011). At the last Olympic Games, Martyna Trajdos explained that being shaken and slapped by her trainer provided the basis for the ritual she put into place to mentally prepare herself before her fights (France, 2021). We might wonder whether this type of athlete would even want help or ever be willing to denounce this type of treatment. In contrast, Marie-José Perec decided at one point to break away from her coach, acknowledging the technical skills she had gained with him that had enabled her to achieve her best performances in the 200 and 400 m, but also noting that she could no longer bear his values, inflexibility, and reprimands (Le Monde, 2003). By better understanding the activities that lead athletes to accept, question, fight, freeze, or flee and reject maltreatment (i.e., negotiate), we might provide more accurate information for building a safeguarding system specifically situated in the elite sports context.

### Psychosocial Interactions as the Lens for Analyzing Sports-Related Maltreatment and Safeguarding Actions

The current safeguarding policies are based on the cognitivist assumption (i.e., a set of theories on the processes of knowledge acquisition) according to which a good practices system will change elite sports behaviors. We argue that believing that sports-related maltreatment can be eradicated is wishful thinking because changing the system will never sufficiently constrain the actions and thoughts of all stakeholders. Coaches, for example, cannot be prevented from using coercive methods to achieve top performances from their athletes, nor can certain athletes be prevented from seeking dependency relationships with their coaches or accepting maltreatment in order to achieve their goals. Thus, we propose to consider sports-related maltreatment at another level: an ecological dynamic approach that assumes that athletes' and coaches' behaviors result from self-organization (as opposed to organizations imposed from schemas, mental models, or cultural influences; Newell, 1991). In other words, even if the sports system must change, it is still important to examine the psychosociological interactions that may organize independently of prevention policies.

In this article, we explore how safeguarding might be expanded to better take into account the complexity of sports participation, especially in what we call the “gray area of coach-athlete relationships.” In this gray area, a wide range of coaching behaviors may be either acceptable or unacceptable (maltreatment) depending on the circumstances, the coach's intent, and the frequency of the maltreatment occurrences. Indeed, the athletes interviewed by Marsollier et al. (2022) described coaching behaviors as unacceptable not based on the nature of the behavior itself but on three main factors: (a) the actual or potential negative consequences for athletes' well-being or performances, (b) the coach's poor ethics (e.g., bad intentions, problematic attitudes, exceeding the role of coach), and (c) the characteristics of the coaching behavior (i.e., uselessness, inappropriateness, with high frequency or high intensity). For example, coaches may hug their athletes to celebrate a victory or console after a defeat (acceptable coaching behavior), but this can slide into sexual abuse if it is done without an athlete's consent. In the same vein, while comparing one athlete to another in a constructive and positive way is acceptable, constantly comparing an athlete in a negative and malicious way would be unacceptable (maltreatment). Also, training at the top level often means mistreating one's own body by pushing it to the limit, whether to gain control or attain forms of narcissist completeness (Lévèque, 1985; Le Breton, 2015; Hauw and Bilard, 2017). Thus, the risk of not examining the behaviors potentially sought or accepted by elite athletes may well result in a naïve understanding of their relationships with the potential maltreatment situations and the associated processes. In this article, we assume that safeguarding is also about equipping sports stakeholders with effective, concrete and good practices (in addition to prevention policies).

### Maltreatment in Elite Sport

A few qualitative studies have highlighted several forms of maltreatment at the top level: apparent forced training with its deleterious physical consequences (Adams and Kavanagh, 2020), yelling, intentional denial of attention and support (Stirling and Kerr, 2008), silent treatment, belittling, scapegoating and isolating (Gervis and Dunn, 2004), as well as rigid weight control regimes and training/competing with injuries (Pinheiro et al., 2014). All these studies have pointed out the gray area of coach-athlete relationships, wherein these athletes negotiate maltreatment situations by mostly accepting them through their normalization. Indeed, despite the negative impact of these behaviors on the interviewed athletes, they normalized them as part of training (Stirling and Kerr, 2009; Pinheiro et al., 2014; Adams and Kavanagh, 2020) and described strategies (Kavanagh et al., 2017) for coping with them. In parallel, a few sociological studies have highlighted the tendency of some athletes to train despite injuries, pain, or exhaustion; the imposition of extreme training regimens; and the negotiation of such situations by normalizing these behaviors in certain sports cultures (Hughes and Coakley, 1991; Kerr, 2010; Pike and Scott, 2015). However, we do not know how the negotiation of maltreatment situations takes place and how it evolves over time and with circumstances: that is, how acceptance is built, whether there is clear consent, and why athletes stay involved even when the situation slips into a conflictual state. Yet not having this knowledge has notably limited our ability to design and build efficient safeguarding policies and procedures for athletes. Additional studies are thus needed to better understand the processes by which athletes negotiate (e.g., accept, reject) the gray-zone sports situations that lead to or become maltreatment. Indeed, we need to gain a more precise understanding of why these situations emerge.

The aims of this article are to (a) present an original approach to describe the evolution in the interactions between athletes and the sports situations that lead to maltreatment (i.e., navigating in the gray area of coach-athlete interactions), (b) identify those alert landmarks that help us recognize the level of risk of athlete maltreatment, and (c) provide concrete guidelines to prevent and deal with sports-related maltreatment.

## Methods

### Paradigmatic Position and Theoretical Approach

In the theoretical framework of the biology of cognition and the autopoietic system (Varela et al., 1974), the situatedness of a phenomenon is linked to human experience and emerges from a self-organized process of coupling that a person establishes with the environment. Situated experience is thus the enactment of the meaningful worlds (i.e., actions, situations, thoughts, emotions, body feelings) in which individuals live, depending on their histories and the current situations and perspectives in which they are engaged (e.g., Varela et al., 1991). For athletes, the sporting environment is generally the main situation: that is, what is meaningful for them at each period of their life course. Indeed, this framework can be used to describe how gray-zone sports situations can slide into maltreatment situations by examining the evolution in the various states of an individual's enacted experience. Moreover, experience is enacted in the various temporal horizons that constitute the dynamics of relationships between past, present, and future time at each meaningful period of an athlete's history (Varela, 1999). This means that experience is a stream or a course represented by the succession of meaningful periods characterized by diachronic and synchronic coherences (e.g., Theureau, 2003). Whereas synchronic coherence describes the structure of participants' activity during a specific episode (i.e., one unit of activity), diachronic coherence provides insight into the successive transformations of participants' activity in their singularity. To summarize, by examining the situatedness of sports-related maltreatment, we can provide descriptions of and insight into the processes by which athletes negotiate (e.g., accept, reject) the gray-zone sports situations that lead to or become maltreatment.

Several sports studies using this situated approach have developed situated analyses in various domains of sporting concerns, such as performance (e.g., Rochat et al., 2018), learning (e.g., Rochat et al., 2020), and coaching (e.g., D'Arripe-Longueville et al., 2001). Notably, studies in the field of the antidoping sciences have investigated the emergence of substance use over athletes' careers and identified the meaningful components of the situations linked to doping (e.g., Hauw, 2013). These studies have identified the diachronic coherence in the succession of life-course episodes that progressively led athletes to accept to begin doping (e.g., Hauw and Bilard, 2012). In addition, the authors have identified the various synchronic organizations that characterize the differentiated meaningful couplings between athletes' activity and doping (e.g., Hauw and Mohamed, 2015). As this research has resulted in prevention recommendations (Hauw, 2017), we expected that the situated approach would offer new insights through an in-depth analysis of the processes by which athletes negotiate the sports situations that lead to maltreatment.

### Participant

Erika (pseudonym), the athlete we interviewed, was a 30-year-old former Canadian boxer who had experienced several forms of maltreatment (i.e., physical and psychological maltreatment, neglect) from her coach for 5 years, until the end of her career. During this period of sporting life, she competed at the national level and practiced 6 days and about 15 h a week. The athlete's experience was collected as part of a global research project to analyze maltreatment situations in high-level sport within the French-speaking world. This project received the approval of the ethics committee of the University of Lausanne 209 (C_SSP_052021_00005).

### Data Collection

As presented in Vignette 1, two types of data were collected to reenact the activity of this athlete who had been maltreated during her career: (a) traces of past activity and (b) verbalizations regarding these traces elicited during an enactive interview.

### Protocol Presentation

This vignette details the protocol of data collection with its three parts: (a) assessment of the athlete's personal resources (stability, support, situation), assurance of or help in setting up a “safe place,” and presentation of the two sessions of data collection; (b) the reconstruction of traces of past activity; and (c) the self-confrontation with these traces of past activity.

#### The Assessment of Personal Resources

The protocol aimed to put the athlete in a situation in which she had to re-enact her past experience. This can be difficult and stressful, and it may generate emotional reactions that need to be controlled. In this part, this is explained to the athlete, as is the researcher's need to ask some personal questions before starting the data collection *per se*. We thus examined the athlete's resources for coping with potentially negative reactions post-interview.

The athlete's ability to handle the interview was assessed on three points: (a) a brief anamnesis to determine her internal and external psychosocial resources (family, professional and possible current difficulties), (b) determination as to whether or not psychological work was or is part of the treatment of one or more of these traumas, and (c) the athlete's affirmation of having a safe place to go as a strategy for managing emotional states.

Generally, if the assessment reveals sufficient resources, the interview continues. “Safe place” training is given in the event of unfamiliarity with the technique. Psychological work in progress postpones the research interview until the work ends. An insufficient number of psychosocial resources (isolation, no social activities) excludes a person from the sample. In this situation, we explain the reason of the decision and thank the participant after asking whether he or she has any further questions.

#### Reconstruction of Traces of Past Activity

The aim of this part is to rebuild the athlete's sporting life history related to the maltreatment with the identification of time periods (i.e., what happened, when, where… until today). It is done with a “factual” interview that usually lasts one and half hours.

The phenomenon under study must be clearly delimited temporally (i.e., ONE form of maltreatment, then other forms of maltreatment: combined forms of maltreatment). The athlete was invited to review past periods of her sporting life course by characterizing meaningful and factual elements that were personal in the development of her sporting life course related to maltreatment. We asked for themes or key events that described a coherent period of the life course (synchronic coherence). To help the athlete and ensure that this coherence was characterized, various themes were systematically asked about, such as types of athlete interactions with coaches, peers, officials, support networks, and medical/paramedical staff. Questions centered on descriptions of facts (e.g., results, hours of training) and actions or behaviors (i.e., What were you doing? What did the coach do?).

**Figure 1:**
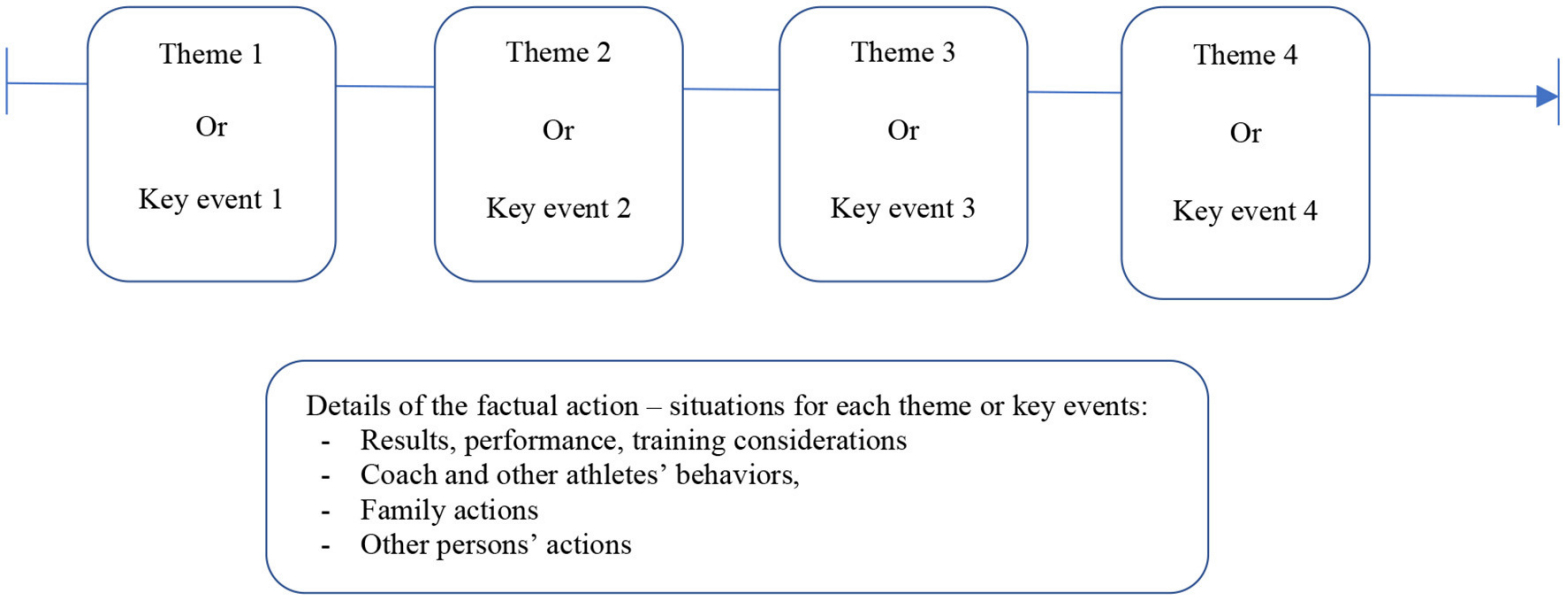
First representation of the sporting life course related to maltreatment.

The interviewer then segmented the sporting life course into coherent, successive episodes. The first representation was structured with the athlete in the form of Figure 1.

This part is aimed at drawing the first representation of the story with a succession of episodes. It is then finalized by the researcher when the first interview is finished, using the transcript (or listening again the recordings).

#### The Self-Confrontation With These Traces of Past Activity

In this part, we ask the athlete to re-enact each of the episodes in order to bring forth the experience linked with these periods. Thus, the presentation of the traces of past activity was displayed and re-read in order to contextualize the activity and stimulate access to the experience. The athlete was asked to concentrate on each episode and relive what was happening. Concretely, we asked her to “try to tell me what you experienced at that time.”

The questions that followed asked for details about emotional reactions, mind states, perceptions of the athlete and her activity. We also sought information on the questions and understandings of what was at stake at this period. We strictly re-centered the athlete on the periods as they were lived and not as she thought about them now. We also asked for probes regarding the changes from one period to another. Finally, requests for ignoring overgeneralization were made (e.g., “training is always hard” or “a coach must be stimulating”).

**Figure 2:**
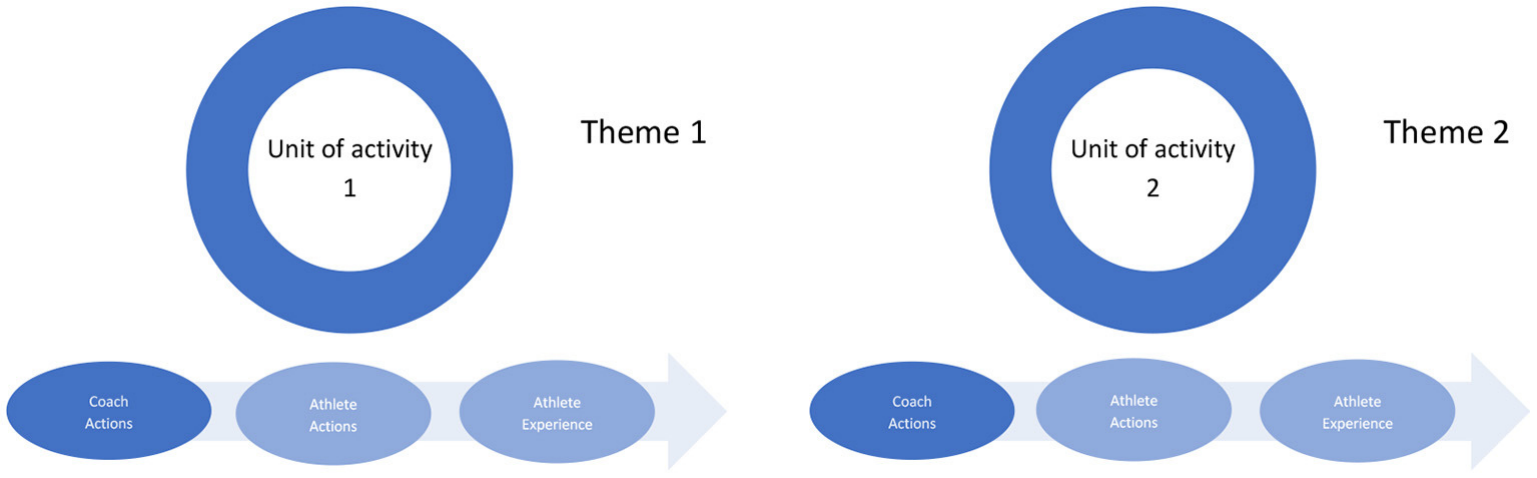
Representation of the diachronic and synchronic coherences of the enacted activity regarding maltreatment.

This part of the interview aims to complete the first representation in two directions: (a) add experiential information regarding each episode, and (b) bring forth the meaningful activity that is included as a whole of factual elements and that is experienced lived by the athlete. This is finalized by the researcher when this second interview is finished, using the transcript (or listening again the recordings) and the theme/key event is moved to the “unit of activity” that corresponds to the meaningful units of meanings in the context of the diachronic and synchronic coherences of the enacted activity regarding maltreatment (see Figure 2). This final representation constitutes the material for data treatment.

We used the data collected during the first interview to organize the sporting life course related to the boxer's maltreatment (see Vignette 1, Figure 1).

### Data Treatment

Following the procedure described in Vignette 1, Figure 2, synchronic and diachronic coherences were first re-built using the transcripts of the first and second interviews. To recall, we began first by considering the results of the first interview, which segmented the timeline into meaningful episodes with themes or key events. Then, for each episode we aggregated the information gathered in the second interview to transform the episodes into units of meaningful activity that the athlete had experienced. Three dimensions were considered: (a) the coach's actions that the athlete perceived as meaningful (e.g., He makes constant remarks about my weight), (b) the athlete's meaningful actions (e.g., I got dehydrated because it was the only way to lose weight), and (c) the meaningful experience related to these first two dimensions (e.g., Every time I put on my gloves, I cry). We used a first- or third-person formulation at this level. Then, we formulated a title that summarized each unit of activity using nouns (e.g., Exhaustion and loneliness, Rebellion). We then independently coded all these data and compared our codings to determine the most representative final version regarding the interviews.

We next examined the elements that described the dynamics of evolution for each of the three dimensions included in the stream of the units of activity. We expected to describe the process by which the interactions between the coach and athlete evolved and the various forms of negotiation regarding maltreatment (e.g., acceptance, rejection). We also expected to identify alert landmarks that would reflect the dynamics of a construction of maltreatment set up by her coach.

## Results

**Figure 3:**
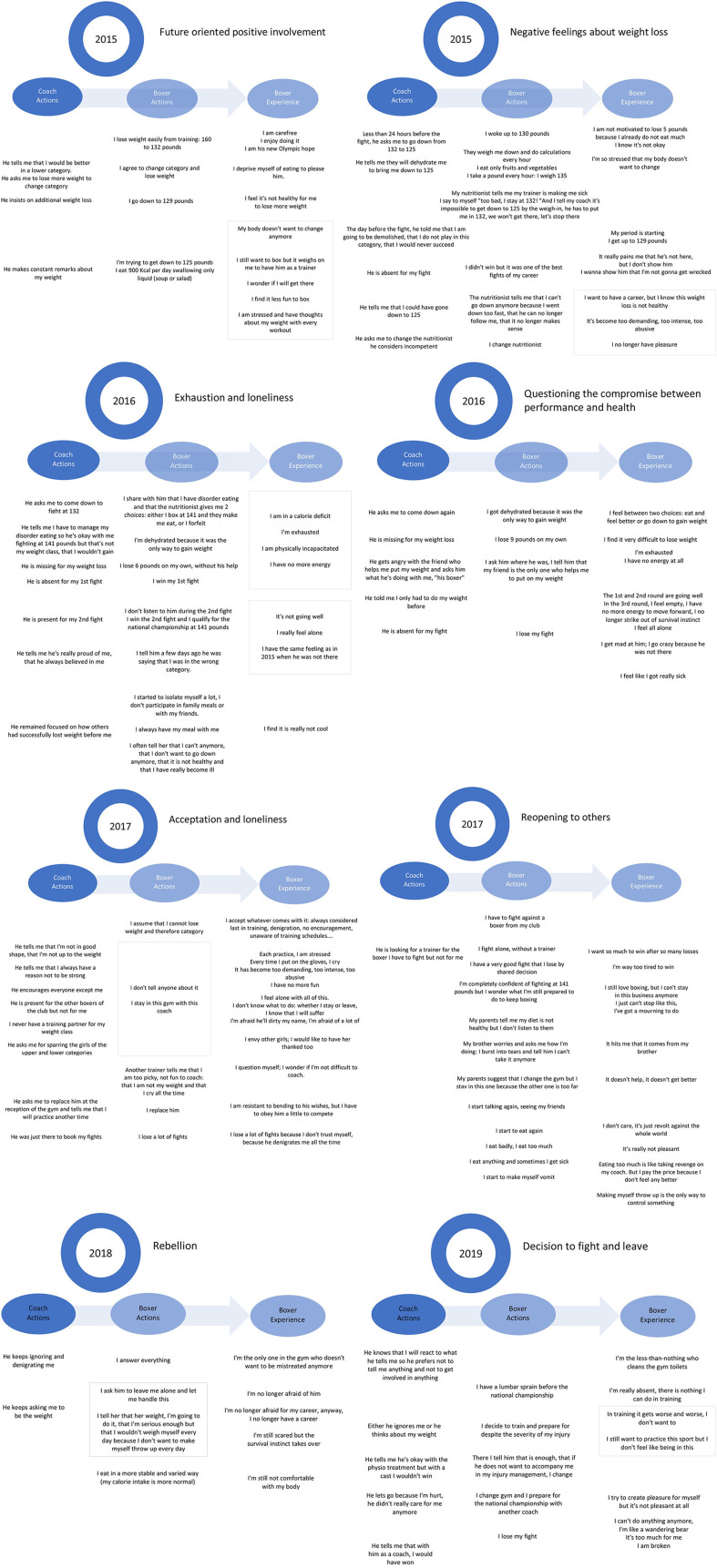
Meaningful boxer ‘slife course and differences in the way of experiencing maltreatment.

In the following sections, we expand on the key elements displayed in Figure 3, which illustrates the methodology for examining the diachronic (i.e., maltreatment experience at one point) and synchronic (i.e., maltreatment experience over the period of time studied) coherences.

The results for the diachronic coherence revealed eight periods that segmented the meaningful life course and the dynamics of acting and experiencing in maltreatment situations. As presented in Figure 3, the boxer shifted from various types of coherence in the interaction with the maltreatment situations. These corresponded to **Acceptance** (i.e., future-oriented positive involvement; Unit of Activity 1), **Regulation attempt** (i.e., negative feelings about weight loss, exhaustion and loneliness, questioning the compromise between performance and health, acceptance and loneliness; Units of Activity 2–5), **Distancing** (i.e., reopening to others; Unit of Activity 6), and **Rejection** (i.e., rebellion and the decision to stand up to her coach and leave; Units of Activity 7, 8).

Figure 3 shows that the synchronic coherence within each subdimension of the units of activity indicated various changes in the athlete's perception of the coach's actions, the athlete's meaningful actions, and the athlete's meaningful experience. We develop each of them in the following section.

First, the **perception of her coach's actions** was characterized by:

(a) the push for high performance (Units of Activity 1–8): Erika's coach was all about performance and the potential for good results in a lower weight category. Each fight required significant weight loss, which was not always achieved. When Erika was over the weight for that category, her coach told her she was going to get demolished in the heavier category, that she would never succeed and never win.

(b) an insistence on changing the weight category (Units of Activity 1–7): Erika's coach insisted on further weight loss by talking about it at every training session and making incessant remarks about her weight. Before each fight, he asked her to lose several pounds within a few hours and made her dehydrate and limit her calorie intake. He went so far as to ask her to change her nutritionist when Erika failed to reach the right weight for a fight.

(c) an inconsistent presence (Units of Activity 2 to 8): Erika's coach was absent for fights for which she had failed to make the weight but reappeared after she won a fight. He also ignored her during most training sessions and favored the other boxers in the gym (e.g., encouraged everyone except her, was present for the other boxers but not for her, never found a sparring partner for her weight category).

and (d) ignoring physical and emotional states (Units of Activity 2–8): Erika's coach knew she had an eating disorder and had been seriously injured but continued to demand that she train and lose weight.

Second, the **athlete's meaningful actions** were directed by:

(a) attempts to lose weight (Units of Activity 1–7): Throughout her career, Erika tried to achieve the weight demanded by her coach by drastically restricting her calorie and fluid intake just before fights, thereby developing eating disorders that affected her performance and wellbeing.

(b) the search for high performance (Units of Activity 1–8): Erika agreed to switch categories and lose weight to perform well enough to have a career in boxing. She qualified for several regional and national competitions and always strove to win her fights.

and (c) the attempt to regulate maltreatment (Units of Activity 2–8): Erika tried to meet the demands of her coach for every qualifying event or fight by attempting to lose weight. When weight loss was not possible or after her coach's absence, she asserted herself and imposed a higher weight category on her coach. She also shared her eating disorders with him and often told him she could no longer lose weight because it was not healthy. She trained and fought alone most of the time and eventually stood up to her coach before changing coaches.

Finally, the results showed that the **athlete's meaningful experience** evolved through nine steps:

(a) a future-oriented positive involvement (Unit of Activity 1): Erika was carefree and had fun boxing and losing weight to change categories; she was her coach's new Olympic prospect.

(b) the emergence of negative emotions toward body feelings and performance (Unit of Activity 1): Erika had less pleasure in boxing and felt that further weight loss was not healthy for her and that she was inflicting it upon herself because she did not love herself.

(c) a general feeling of distress (Units of Activity 1, 2): Erika felt an increasingly anxiogenic, stressful training climate: incessant thoughts about her weight occurred at every training session and she felt stressed that her body did not want to change (i.e., lose weight).

(d) a state of survival (Units of Activity 3, 4): Erika experienced calorie deficit, exhaustion, physical incapacity, feelings of loneliness, and the feeling that she was really sick.

(e) questions about the compromise between performance and health (Unit of Activity 4): Erika wondered about her ability to lose weight and perform well, as well as about her pleasure/motivation for boxing.

(f) a stressful acceptance (Unit of Activity 5): Erika agreed to always be the last in training, to be denigrated, to not be encouraged, to not have a coach present during training… Her coach had become too demanding, too intense, too abusive.

(g) an emotional and social reaction (Units of Activity 5, 6): Erika was jealous of the girls her coach was taking care of and felt alone without knowing what to do; she cut herself off from the other athletes, her family and her friends.

(h) an attempt at distancing from stressors (Units of Activity 6, 7): Erika rebelled and was the only one in the gym who no longer accepted being mistreated; she ended up no longer being afraid for her career, which she felt was over.

and (i) being in a broken state (Unit of Activity 8): Greatly diminished by her injury, Erika was completely absent from training; she could no longer do anything, and during her last fight she was not even able to fight or defend herself; she felt broken.

## Discussion

Our study revealed the evolutive process of maltreatment in eight units of activity. This evolution describes a dynamic change in the way the boxer negotiated gray-zone sports situations that slid into maltreatment through (a) Acceptance (i.e., the future-oriented positive involvement), (b) Regulation attempt (i.e., negative feelings about weight loss, exhaustion and loneliness, questions about the compromise between performance and health, acceptance and loneliness), (c) Distancing (i.e., the reopening to others) and (d) Rejection (i.e., rebellion and the decision to stand up to her coach and leave). Indeed, the way the boxer shifted from the various types of synchronic coherence in her interactions with the maltreatment situations revealed four alert landmarks in the dynamics of a maltreated athlete's activity.

In this section, we discuss each alert landmark in line with the current literature and in terms of prevention as a concrete safeguarding intervention.

**Acceptance:** First, during her future-oriented positive involvement, Erika was carefree and had fun boxing and losing weight to change categories; she was her coach's new Olympic prospect. Her actions during this time were centered on trying to achieve the weight demanded by her coach, who was expecting high performances and good results in a lower weight category. Listening to the coach without asking questions suggests the notion of plausibility described by Smits et al. (2017). Indeed, plausibility provides an explanation for Erika's faith and trust in her coach, to such an extent that she did not think critically about what the coach was asking of her. Smits et al. suggested that the young age of athletes would explain their passivity and obedience, yet this might be questioned here as our study concerned an adult athlete. We might therefore explain the boxer's obedience by the coach's power over her – more precisely, his legitimate authority, expertise and past successes (Stirling and Kerr, 2009). It is also interesting to question this athlete's relative credulity. She seemed to accept the coach's injunctions without question, which is a sensitive aspect of athlete-coach interactions that should be taken into account for an informed sporting practice (Gamble, 2021).

### Concrete Situated Safeguarding Actions: Importance of Supporting Athletes to Develop an Enlightened Awareness of What They Decide and/or Agree to Do

The athlete's lack of power seemed to drag her into a situation of abuse: she was directed to switch categories without discussion or explanation. In addition, this maltreatment situation was especially hard to identify as such because weight loss “is part of” her sport. In terms of safeguarding actions, it appears essential to determine an athlete's commitment to the sports situation, both rational commitment (motivation and associated behaviors) and emotional commitment (less conscious, limbic and emotional brain) (e.g., Peters, 2021). Thus, the notion of consciousness appears primordial: the athlete's acceptance of a sports situation with an awareness of what it implies (i.e., enlightened acceptance). Sports psychologists and mental performance consultants appear to be essential professionals, as they are skilled at identifying both the rational and emotional commitment of high-level athletes and can thus help them develop a keener awareness of the types of situations they are facing.

#### Regulation Attempt - Dealing With Maltreatment

Second, Erika felt that further weight loss was unhealthy, but her actions were geared toward striving for high performance to the detriment of her mental and physical integrity. In parallel, her coach insisted that she change her weight category despite her incapacity to do so. Erika felt an increasingly anxiogenic, stressful training climate and had incessant thoughts about her weight in every training session and high stress because her body did not want to change (i.e., lose weight). After attempting to regulate with her coach (e.g., choosing the weight category after trying to lose weight, changing nutritionists when he asked her to), she began to regulate alone and isolated herself in order to find a solution on her own (e.g., not listening to her coach during a fight, isolating from others, losing weight alone). This showed how a normal sports situation turned into maltreatment. Indeed, the problem was not simply the demand for weight loss *per se*, which is a mistreatment, but it was compounded by the lack of support and guidance in losing the weight. Later in the process, the coach's refusal to acknowledge her inability to lose further weight, his negative and mean comments, and his ignorance about and emphasis on weight loss put Erika deeper into a situation of maltreatment. These abusive coaching behaviors have been identified in other qualitative studies: degrading comments, intentional denial of attention and support (Stirling and Kerr, 2008); criticism and “silent treatment” (Stirling and Kerr, 2009); and rejection, ignoring and isolating (Gervis and Dunn, 2004). While some studies suggest that abused athletes seek support (e.g., Kavanagh, 2014; Kavanagh et al., 2017), the boxer we interviewed tried to regulate her interactions with maltreatment with her coach, then on her own. As her coach isolated her from the rest of the group, she gradually began to isolate herself from her coach, her training partners, and her friends and family. Last, Stirling and Kerr (2009) found that a coach's power with regard to an athlete's experience of maltreatment can induce fear, which then influences the athlete's ability to report the abuse. In the present study, the boxer was afraid for her career. To summarize, our results suggest how a normal sports situation can become a situation of maltreatment because of (a) no support, (b) insistence, and (c) negative supervision.

### Concrete Situated Safeguarding Actions: Technical and Emotional Support for Athletes

Erika felt that her coach was forcing her into unreasonable and unhealthy behaviors, and the emotions associated with this situation quickly changed from motivation and recklessness to negative emotions about herself and her performance. Thus, the Acceptance and Regulation attempt phases seem to be key stages in which sports stakeholders must be vigilant, questioning the athletes about the decisions imposed on them vs. the choices proposed to them. Also, they must ensure that the coach and/or technical and medical staff have put into place an appropriate framework. For example, the coach and medical staff should technically and emotionally support the athletes and demonstrate patience and kindness as they pursue their goals.

#### Distancing

Third, Erika realized that boxing in these conditions was unhealthy for her but did not feel ready to stop. Her family's comments about her diet made her react and reopen from a social point of view (she came out of isolation). This unit appeared when the situation was very much degraded. It offered a kind of salutary bifurcation that emerged when the degradation became too strong and she felt the need for a support network. Without these two parameters, the situation could have drifted in a more worrisome way for her health.

### Concrete Situated Safeguarding Actions: Support and Supervision Adapted to the Specific Requirements of Elite Sport, and Acting as a Safeguard vis-à-vis an Abusive Coach if Necessary

Given the many requirements for success in sports, it can be difficult to distinguish a normal sporting situation from a situation of maltreatment. It therefore may be useful to surround elite athletes with a sports network that can (a) offer support and supervision adapted to the specific requirements of elite sport and (b) if necessary, act as a safeguard vis-à-vis an abusive coach. Thus, until prevention policies succeed in putting abusive coaches out of the system, it seems important to offer support to athletes, as well as concrete directives to members of both the technical (e.g., physical trainers, osteopaths, mental performance consultants, nutritionists) and medical (e.g., doctors, sports psychologists) staff. These professionals must be able to detect the maltreatment situations in which athletes may find themselves without yet being fully aware of them. Again, sports psychologists and mental performance consultants appear to be key players, given the ethical principles that guide their practice, their proximity to the athletes, and the trust they have built with them.

#### Rejection

Finally, Erika asserted herself and decided to leave her coach when he refused to adjust her training to take into account an injury. Stirling and Kerr (2009) retrospectively examined elite female swimmers' experiences of emotional abuse and identified three stages: early sports career (normalization and acceptance of maltreatment), mid sports career (acceptance of maltreatment if the athlete is performing well), and late sports career (rebellion, questioning the coach). The present analysis of the dynamic changes in the interactions between the boxer and the maltreatment situations revealed a more complex way an athlete can accept, regulate and reject a maltreatment situation. In parallel, Stafford et al. (2013) found that being forced to continue to train when injured or exhausted is by far the most common of the physically harmful behaviors. Interestingly, the present case revealed that the boxer made the decision to train and compete while injured and exhausted. Although she rejected the maltreatment situation she had been interacting with for several years, she still decided to mistreat herself.

### Concrete Situated Safeguarding Actions: Question and Accompany the End or Continuation of the Elite Sports Career

At this level, the aim of safeguarding interventions is to help athletes deal with the maltreatment experience, even to take advantage of it for growth. The question is: how can athletes be helped to overcome this experience? At this stage of the maltreatment experience, it is important to question the athletes about their motivations for continuing in their sport and to support them in their decision to either end or continue their careers. Indeed, the end-of-athletic-career stage can provoke feelings of loss and void in elite athletes (Stephan, 2003). For this reason, they need clear retirement plans that will foster favorable emotions and coping behaviors during the transition (Alfermann, 2000; Stambulova et al., 2007), as well as ongoing support for positive reinterpretations of their retirement (Dimoula et al., 2013). At the minimum, a consultation with a sports psychologist or mental performance consultant should be scheduled. In the case of a return to sport following an injury, anxiety symptoms might be reduced through cognitive behavioral therapy or eye movement desensitization and reprocessing interventions, with the return to competition secured through the retreatment of the emotional, cognitive and body dysfunctions (e.g., Shapiro, 2017). This is particularly relevant in situations of the sports-related maltreatment experience, given its traumatic aspect (Mountjoy et al., 2022; Tuakli-Wosornu et al., 2022). It is also important to consider the potential for growth (e.g., awareness of the power of decision and action in a maltreatment situation) and, when applicable, psychological interventions could be designed in this direction, such as those proposed by Peters (2021).

In summary, our results offer situated safeguarding implications for supporting and guiding elite athletes and their coaches through the gray area of coach-athlete relationships in order to enable them to (a) avoid maltreatment situations and (b) help them extricate themselves before this situation becomes a danger. Indeed, our results revealed four progressive alert landmarks that situate the athlete in this gray area of the coach-athlete relationship versus full-blown maltreatment, along with a “timeline” of maltreatment escalation. These key warnings are as follows: (a) Acceptance, (b) Regulation attempt, (c) Distancing, and (d) Rejection. We believe that these alert landmarks will enable a finer-grained diagnosis of developments in the gray area of coach-athlete relationships that could be used by sport psychologists and mental performance consultants to support athletes through better targeting of the right actions. These professionals might thus provide (a) guidance to ensure that they develop an enlightened awareness of what they are deciding and/or agreeing to do, (b) technical and emotional support, (c) support and supervision adapted to the specific requirements of elite sport and actions that serve as safeguards vis-à-vis an abusive coach if necessary, and (d) a line of questioning that can help the athletes decide whether to end or continue the elite sports career and accompaniment in carrying out the decision. For example, these alert landmarks could prompt sports federations to better oversee coach-athlete relationships or help existing hotlines to receive testimony from whistleblowers. Indeed, the concrete actions associated with each alert landmark could guide sport psychologists, mental performance consultants, and sports federation hotline responders to better respond to athletes' potential and actual maltreatment experiences, as has been done in the doping domain (e.g., Bilard et al., 2011; Mohamed et al., 2013; Hauw, 2017).

Some limitations should be noted, especially the difficulties and challenges of retrospective designs. Also, we acknowledge the limitations due to the process of building new meaning when an athlete is explaining his or her past maltreatment history. However, our original method, derived from the enactive and situated paradigms, offers several possibilities to limit the weaknesses of traditional verbal reporting (Hauw and Bilard, 2012). Further research should be conducted to identify, compare, and generalize knowledge on all the mechanisms that lead an athlete to accept or reject maltreatment in a sporting life course.

## Conclusion

The main results of our study are the concrete safeguarding guidelines that (a) may prevent sports-related maltreatment and (b) provide new insights into the gray area of this type of maltreatment. We identified four progressive alert landmarks – or key warnings (i.e., Acceptance, Regulation attempt, Distancing, Rejection) – that point to a “timeline” of maltreatment escalation. These alert landmarks provide concrete information to both researchers and sports stakeholders on the negotiation stages that maltreated elite athletes may pass through. In addition, these alert landmarks constitute an effective observation grid that can help the stakeholders surrounding athletes (e.g., doctors, mental performance consultants, sports psychologists) to remain vigilant as to possible tipping in a maltreatment situation and to surround maltreated athletes when necessary. The methodology we developed within the enactive framework allowed us to account for the dynamics of (a) the generation of an experience of maltreatment, (b) the interactions between the experiences of maltreatment and athletes' activity, and (c) the development of these experiences at different time scales. Given the potential of this approach to provide insights into the dynamics at work in the gray area of coach-athlete interactions, our case study calls for future studies in sports with different requirements (e.g., endurance, artistic, team sports). It also suggests the interest of examining the maltreatment situation negotiations of several athletes within the same study. Another avenue of study might be to have the medical staff members surrounding elite athletes (e.g., doctor, mental performance consultant, sport psychologist, etc.) test these four stages of alert to determine their advantages and difficulties.

To sum up, this original methodological approach offers a description and analysis of the nature of sports-related maltreatment. It therefore broadens our understanding of this phenomenon and suggests safeguarding actions that complement the safety-oriented policies already in place. Indeed, our pragmatic perspective has increased knowledge on how to better help elite athletes, guide coaches more precisely in their practices, and equip the many technical and medical staff members.

## Data Availability Statement

The original contributions presented in the study are included in the article/supplementary material, further inquiries can be directed to the corresponding author.

## Ethics Statement

The studies involving human participants were reviewed and approved by Research Ethics Commission of the University of Lausanne (CER-UNIL). The patients/participants provided their written informed consent to participate in this study.

## Author Contributions

ÉM and DH thought about the project together and participated in all stages of data collection and analysis, writing and review of the article. Both authors contributed to the article and approved the submitted version.

## Funding

Open access funding provided by University of Lausanne.

## Conflict of Interest

The authors declare that the research was conducted in the absence of any commercial or financial relationships that could be construed as a potential conflict of interest.

## Publisher's Note

All claims expressed in this article are solely those of the authors and do not necessarily represent those of their affiliated organizations, or those of the publisher, the editors and the reviewers. Any product that may be evaluated in this article, or claim that may be made by its manufacturer, is not guaranteed or endorsed by the publisher.
